# Investigating Nutritional and Inflammatory Status as Predictive Biomarkers in Oligoreccurent Prostate Cancer—A RADIOSA Trial Preliminary Analysis

**DOI:** 10.3390/nu15214583

**Published:** 2023-10-28

**Authors:** Mattia Zaffaroni, Maria Giulia Vincini, Giulia Corrao, Chiara Lorubbio, Ilaria Repetti, Federico Mastroleo, Costantino Putzu, Riccardo Villa, Sofia Netti, Oriana D’Ecclesiis, Stefano Luzzago, Francesco Alessandro Mistretta, Gennaro Musi, Federica Cattani, Sara Gandini, Giulia Marvaso, Barbara Alicja Jereczek-Fossa

**Affiliations:** 1 Division of Radiation Oncology, IEO European Institute of Oncology IRCCS, 20141 Milan, Italymariagiulia.vincini@ieo.it (M.G.V.); giulia.corrao@ieo.it (G.C.); ilaria.repetti@ieo.it (I.R.); federico.mastroleo@ieo.it (F.M.); costantinoputzu@icloud.com (C.P.); riccardo.villa@ieo.it (R.V.); giulia.marvaso@ieo.it (G.M.); barbara.jereczek@ieo.it (B.A.J.-F.); 2 Department of Oncology and Hemato-Oncology, University of Milan, 20122 Milan, Italy; stefano.luzzago@ieo.it (S.L.); francescoalessandro.mistretta@ieo.it (F.A.M.); gennaro.musi@ieo.it (G.M.); 3 Department of Translational Medicine, University of Piemonte Orientale, 20188 Novara, Italy; 4 Department of Experimental Oncology, IEO European Institute of Oncology IRCCS, 20141 Milan, Italy; sofia.netti@ieo.it (S.N.); oriana.decclesiis@ieo.it (O.D.); sara.gandini@ieo.it (S.G.); 5 Division of Urology, IEO European Institute of Oncology IRCCS, 20141 Milan, Italy; 6 Medical Physics Unit, IEO European Institute of Oncology IRCCS, 20141 Milan, Italy; federica.cattani@ieo.it

**Keywords:** oligometastatic disease, biomarkers, inflammation, nutritional status, prostate cancer, quality of life

## Abstract

(1) Background: In the RADIOSA phase II randomized clinical trial (NCT03940235), the biology task entails the identification of predictive and prognostic biomarkers in the context of oligorecurrent, castration-sensitive prostate cancer in order to distinguish polymetastatic from oligometastatic disease. This may lay the groundwork for personalized treatments for those patients who could really benefit from metastasis-directed therapies. (2) Methods: Oligorecurrent PCa pts with three or fewer bone or lymph nodal localizations were randomized 1:1 to receive SBRT alone (arm A) or SBRT + 6 months of ADT (arm B). Common serum-derived biomarkers were collected at baseline, and at 3 months after RT. The prognostic nutritional index, an immune and nutrition-based prognostic score, and the controlling nutritional status (CONUT) score, a scoring system for evaluating patient’s nutritional status, were calculated in accordance with the body of available literature. As inflammatory indicators, neutrophil–lymphocyte ratio (NLR) and the NLR–albumin ratio (NLRAR) were assessed. Changes in these parameters between baseline and the 3-month timepoint were evaluated both in absolute and relative values. Changes in these parameters between baseline and the 3-month timepoint were evaluated. Significant differences in the trend of these parameters were assessed using the non-parametric Wilcoxon rank-sum test. A network analysis to analyze the relationships between different features stratifying patients according to the arm of study and site of metastases was performed. (3) Results: The current analysis comprised 88 patients (45 arm A, SBRT only, and 43 arm B, SBRT + ADT). When patients were stratified by ADT administration, cholesterol values showed an increasing trend in the group receiving ADT (*p* = 0.005) which was no longer significant at 1 year. When patients were stratified by site of metastases (52 lymph nodal, 29 bone localizations), the value of NLR was found to be increased in patients with bone localizations (*p* < 0.05). In addition, the network analysis showed that BMI and NRI are strongly and directly linked for patients at baseline and that this correlation is no longer found at three months. Finally, when patients were divided according to time from surgery to oligorecurrence (enrollment) the patients with a longer time (>6.7 years) showed an increase in CONUT score from baseline. All the other nutritional and inflammatory scores or parameters investigated in the present analysis showed no statistically significant differences at baseline, three months, 1 year, and in absolute change. (4) Conclusions: The nutritional and inflammatory parameters do not seem to represent valuable candidates for possible use in clinical decision making in our cohort of patients and a reliable biological characterization of the oligometastatic state in prostate cancer still seems far from being achieved. Ongoing molecular analysis will show if there is a role of mutational landscape in the definition of the oligometastatic state.

## 1. Introduction

The use of metastasis-directed therapy (MDT) is rapidly increasing in the setting of oligometastatic prostate cancer (PCa) [1,2,3]. Radiotherapy and in particular stereotactic body radiation therapy (SBRT) represents a low-toxicity treatment for PCa localizations, with accumulating evidence suggesting that local treatments could defer disease progression, delay the need for systemic and hormonal therapy (androgen deprivation therapy, or ADT), and consequently their related toxicities [1,2,3,4,5]. 

However, in some cases, clinical oligometastasis may represent only the tip of the iceberg for a subclinical polymetastatic disease. In this scenario, proper patient selection, as well as the definitions of the real oligometastatic state, may be of critical importance to properly guide the treatment choice in the oligometastatic disease. While important advances in prostate-specific membrane antigen—positron emission tomography (PSMA-PET) and in advanced imaging overall have been reached [6,7], what is currently lacking, despite preliminary efforts in this direction [8], is a proper biological characterization of the oligometastatic PCa [9,10,11].

It is well known that nutrition and immunity play an important role in cancer progression and in the metastasisation process [12] and inflammatory cells and immune response have consistently been recognized as important factors in the prognosis of cancer [13,14]. Peripheral blood cells, including lymphocytes, monocytes, neutrophils, and platelets have been widely reported in previous research as promoting tumor proliferation and invasion [15,16]. Thus, based on this evidence, many combinations of inflammatory indices such as the neutrophil-to-lymphocyte ratio (NLR), platelet-to-lymphocyte ratio (PLR), and the systemic immune-inflammation index [(platelet × neutrophil)/lymphocyte] have been used to predict PCa prognosis [17,18,19]. In this regard, several studies have identified a new inflammation index called hemoglobin–albumin–lymphocyte–platelet index (HALP), which has proven to be a good prognostic indicator in metastatic PCa [20] as well as gastric, colorectal, renal, and bladder cancers [21,22,23,24]. 

In parallel, patients’ nutritional status (including albumin levels, hemoglobin, and other nutritional indexes) has also been indicated as an important factor affecting oncological outcomes [25,26,27,28]. In particular, Prognostic Nutritional Index (PNI) has shown prognostic advantages in metastatic prostate cancer [26] and similarly the Nutrition Risk Index (NRI) developed by Buzby et al. [29] has proven to be a reliable screening tool for surgical patients, helping to stratify patients preoperatively based on their risk of complications. Importantly, the NRI has been shown to be an independent prognostic factor for several cancer types [30,31,32]. 

PCa patients’ nutritional status has been demonstrated to be particularly affected by ADT administration which is linked to an increase in cholesterol and triglyceride levels, increased visceral fat, decreased testosterone levels, and a decrease in glucose tolerance [33,34,35]. According to the international guidelines ADT is used in patients with intermediate- or high-risk localized PCa and in recurrent or metastatic scenarios, with about 50% of all PCa patients receiving ADT at some point during the course of their disease [36]. Although it represents a relatively well-tolerated therapy, it can cause adverse events that can have a negative impact on patients’ quality of life and consequently impair treatment compliance [35,37].

The aim of the present study is to report preliminary results of the biology task of the phase II randomized clinical trial RADIOSA (NCT03940235) [38]. The biology task entails the identification of predictive and prognostic biomarkers in the context of oligorecurrent, castration-sensitive prostate cancer in order to distinguish polymetastatic from oligometastatic disease. In the present work, different nutritional and inflammatory indexes will be analyzed in order to evaluate both their capability to discriminate treatment outcomes and their ability to discern between the true oligometastatic patients and those who are going to develop a high burden of disease. In addition, the impact on patients’ quality of life of a short course of ADT will be investigated. This may lay the groundwork for personalized treatments for those patients who could really benefit from MDTs.

## 2. Materials and Methods

### 2.1. Study Design

**Figure 1:**
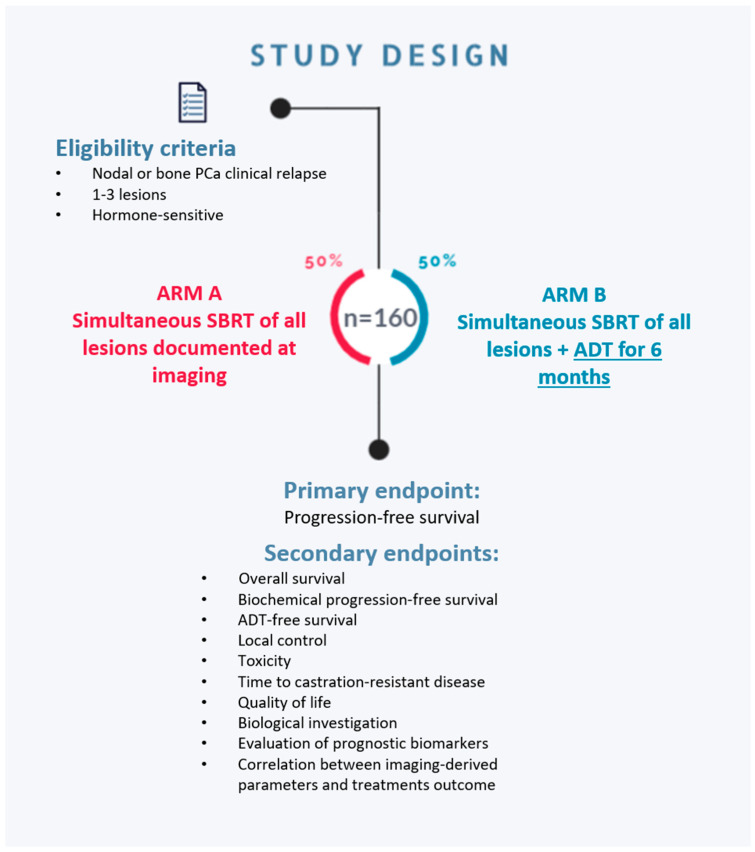
Overview of the RADIOSA trial study design. Abbreviations: PCa (prostate cancer); SBRT (stereotactic-body radiation therapy); ADT (hormonal therapy).

The present study represents a preliminary report of the results of the ongoing RADIOSA trial [38], a phase II randomized study. The RADIOSA trial enrolls patients with oligorecurrent PCa with bone or lymph node localizations (with up to 3 lesions) after curative treatment on the primary tumor. An overview of the trial design is reported in Figure 1. 

Patients enrolled in the trial are randomized into two treatment arms:

- Arm A—MDT with stereotactic radiotherapy on all metastatic sites;
- Arm B—MDT with stereotactic radiotherapy on all metastatic sites + 6 months of ADT.

The study (NCT03940235, AIRC IG-22159) [38,39] started in September 2019 and, at the time of the present study, 105 patients have been enrolled. 

### 2.2. Study Participants

Patients enrolled within the ongoing RADIOSA trial and with a minimum follow-up of six months were considered for study inclusion. All patients were stratified into two treatment arms according to the administration of ADT and the location of the metastases. This trial had been approved by the Ethics Committee of the IEO and Centro Cardiologico Monzino (IEO UID 997). All patients signed a dedicated informed consent before trial enrollment.

### 2.3. Treatment Characteristics

All patients enrolled received a SBRT schedule of 30 Gy in 3 fractions every other day or equivalent regimens (Biologically Effective Dose (BED) > 100 Gy with an α/β  =  1.5 Gy) depending on disease site location. Patients in arm B received 6-months ADT (luteinizing hormone-releasing hormone (LHRH) agonist or antagonist) starting within 1 week before the SBRT treatment.

### 2.4. Data Collection

As per protocol, blood samples of the patients were collected at randomization, at 3 months from completion of the radiant treatment, and at the eventual clinical relapse. All the samples were stored in the European Institute of Oncology (IEO, Milan, Italy) Biobank.

Hematological parameters, including neutrophils, lymphocytes, platelets, hemoglobin, cholesterol, albumin, glycemia, lactate dehydrogenase (LDH), and body mass index (BMI) were collected at baseline. Moreover, common serum-derived biomarkers collected at different time points were considered as standalone biomarkers and together for the calculation of different nutritional and inflammatory indexes. In particular, nutritional status was evaluated through the following indexes:

Controlling nutritional status (CONUT) score [40];

- PNI [26];
- NRI [30];

while considered inflammatory scores were:

- NLR [41];
- NLR–albumin ratio (NLRAR) [42];
- HALP score [20];
- PLR score [41].

### 2.5. Data Analysis

Cholesterol, albumin, glycemia, LDH, NLR, NLRAR, HALP, PLR, PNI, and NRI values at baseline and 3 months after SBRT were evaluated grouping patients according to ADT administration (ARM A vs. ARM B), biochemical recurrence (BCR) (yes vs. no), and site of metastatses (at least one bone lesion vs. lymphnode(s) only). Additionally, cholesterol, glycemia, testosterone, and LDH at 1 year were evaluated grouping patients according to ADT administration (ARM A vs. ARM B). 

Changes in these parameters between baseline and 3 months/1 year after SBRT were evaluated and significant differences in these parameters according the above-mentioned stratifications were assessed using the non-parametric Wilcoxon rank-sum test (Mann–Whitney *U* test). 

To identify possible prognostic biomarkers, differences in the considered parameters were investigated at baseline, also, grouping patients according to the presence of BCR. Furthermore, patients were divided according to quartiles of the distribution of the time from surgery to oligorecurrence to assess any impact of this interval on the change in CONUT score. 

Correlations among biomarkers are measured through Spearman correlation coefficients, which are “joined” in a graphical representation to create a network, both at baseline and 3 months after RT, stratifying patients according to treatment arm and site of metastases. In the network, nodes represent individual biomarkers, while Spearman correlation coefficients between biomarkers are depicted as edges (lines connecting nodes). The color spectrum of the edges ranges from green (direct correlation) to orange (inverse correlation). The thickness of the edges indicates the strength of the association. The position of a node reflects its centrality or rather the importance of a node within the network. All the analysis were performed with R Studio software (R version 4.1.1 R Core Team (2020). R: A language and environment for statistical computing. R Foundation for Statistical Computing, Vienna, Austria).

## 3. Results

A total of 88 patients were included in the present analysis (45 arm A SBRT only, 43 arm B, SBRT + ADT). Median time from last active treatment to enrollment was 36 months (IQR 17–64) with a median age at enrollment of 69 years (IQR 64–75). A total of 56 patients had lymphnodal lesion(s) only, while 32 had at least one bone lesion. 

**Figure 2:**
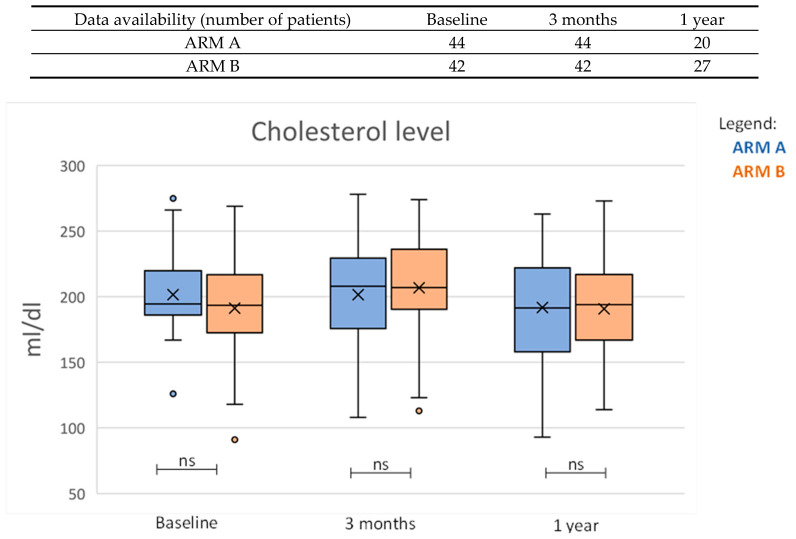
Absolute change in cholesterol levels divided by treatment arms at baseline, three months, and one year. Abbreviations: ns (difference not statistically significant).

Patients’ baseline characteristics, for the whole cohort and according treatment arm, are reported in Table 1. When patients were stratified by ADT administration (Table 2), cholesterol values showed an increasing trend from baseline to 3 months FU in the group receiving ADT (*p* = 0.005), and the change in albumin level was also different between the 2 groups (*p* = 0.02444). Median increase in cholesterol levels in the group receiving ADT was 8% (IQR 1–17%). All the other investigated parameters showed no statistically significant differences at baseline, three months, and in absolute change. At 1 year the change in cholesterol values was no longer significant, indicating a recovery to normal cholesterol values for most patients who received ADT (Figure 2). The 1-year change for the additional investigated parameters, namely glycemia, testosterone, and LDH was not statistically significant (Table 3). 

When patients were divided according to BCR, no significant differences were found in the considered parameters at baseline between the two groups. More detailed results regarding the oncological outcome will be published separately and are not the object of this paper.

When patients were stratified by site of metastases (56 lymph nodal, 32 bone localizations) (Table 4), the value of NLR and NLAR were found to be increased in patients with bone localizations (*p* = 0.0198) at 3 months after SBRT. In addition, an increasing trend with borderline significance (*p* = 0.06) was observed for PLR values in patients with bone localizations.

**Figure 3:**
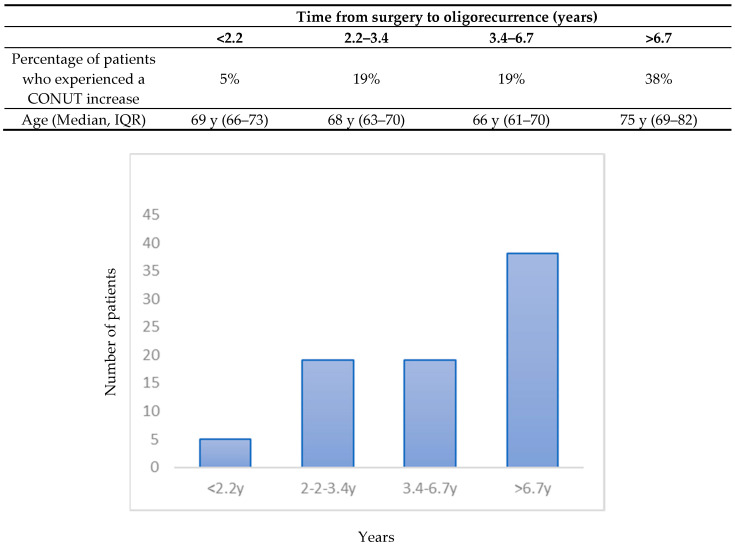
Percentage of patients who experienced an increase in the CONUT value (from baseline to 3 months) according to quartiles of time from surgery to oligorecurrence. Abbreviations: CONUT (Controlling nutritional status); y (years).

Interestingly, when patients were divided into quartiles according to the time from surgery to oligorecurrence (Figure 3), 38% of patients in the fourth quartile experienced an increase from baseline to 3 months in the value of CONUT, depicting a worsening in the nutritional status for these patients.

No significative change among the other investigated biomarkers was found when patients were stratified according to treatment arm, lesion(s) site or BCR. 

**Figure 4:**
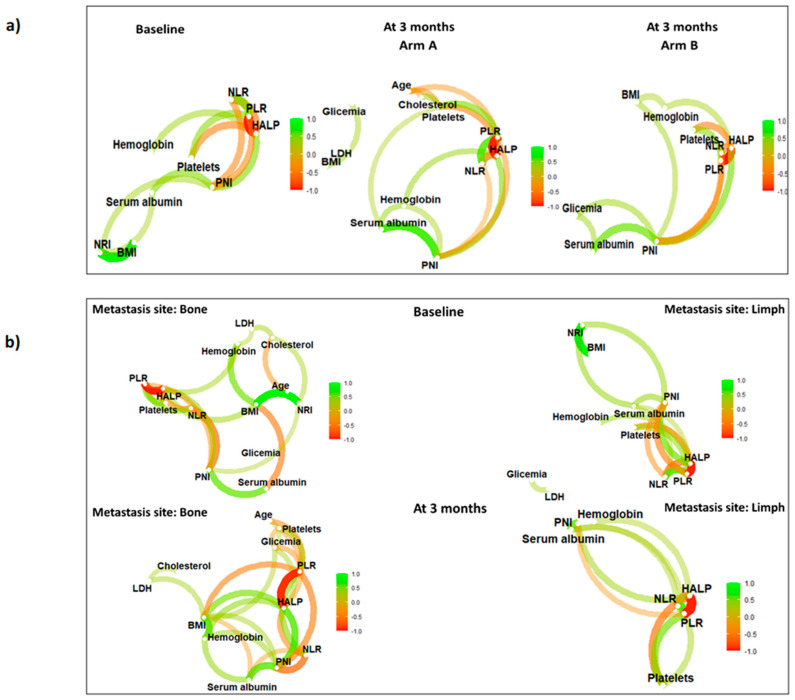
Network analysis for the investigated parameters stratified by treatment arm (**a**) and site of metastases (**b**) at baseline and three months after treatment. In the network, nodes represent individual biomarkers, while Spearman correlation coefficients between biomarkers are depicted as edges (lines connecting nodes). The color spectrum indicates direct (green) or inverse (orange) correlation (1 = maximum direct correlation, −1 = maximum inverse correlation) while the edges thickness indicates the strength of the association. Abbreviations: BMI (Body mass index); HALP (Hemoglobin-albumin-lymphocyte-platelet index); LDH (Lactate dehydrogenase); (NLR) Neutrophil-to-lymphocyte ratio; NLRAR (NLR–albumin ratio); (NRI) Nutrition Risk Index; PLR (Platelet-to-lymphocyte ratio); PNI (Prognostic Nutritional Index).

Figure 4 reports the results of the network analysis for all the considered parameters stratifying patients according to both ADT administration and site of metastasis. To be noted is that BMI and NRI baseline result strongly and directly correlated at baseline for all the patients and that this correlation is no longer present at three months both when patients are stratified according to the treatment arm and to the site of metastases. No other clinically relevant correlation was found in our population for the investigated variables.

## 4. Discussion

The purpose of the present study is to report preliminary data about candidate biomarkers in oligorecurrent PCa representing the nutritional and inflammatory status. No valuable biomarkers related to the nutritional and inflammatory status able to help in oligometastatic Pca patients stratification were found in the present analysis. These results reflect the current scientific literature on the topic where there appears to be a paucity of biomarkers able to assess specific time points or different settings of PCa development [9,43].

Nevertheless, we were able to report evidence that the addition of ADT appears to have an impact on cholesterol and albumin levels, two markers of deteriorating quality of life. In line with the present results, reporting a median increase of 8% for cholesterol levels, several studies showed a mean increase of 3.2–10.6% in total cholesterol with the duration of ADT ranging from 24 weeks to 12 months in the first 3 or 6 months of ADT [44]. A recent study by Fowler et al. [45] reported that PCa patients treated with ADT showed a statistically significant reduction in different quality of life items, such as mental health and general health, energy, and concern regarding body image. In addition, Tucci et al. [35] revealed that fatigue and depressive disorder induced by ADT can interfere with cognitive function, producing a significant reduction in the quality of life of patients with prostate cancer. The concept of ADT-free survival, intended as a delayed start of systemic therapy, has emerged, in particular in the oligometastatic setting as a way to delay the negative side effects of the treatment, such as the increased occurrence of cardiovascular events and metabolic syndrome [46]. The aim of the RADIOSA trial and of MDTs, in general, is to achieve local control and delay progression, and thereby postpone the need for further treatment as ADT. In our study a short course of 6 months ADT resulted in a significant increase in cholesterol levels, in line with the available evidence, nevertheless, the cholesterol levels returned to normal levels at 1 year. How this change may have affected the quality of life of patients undergoing hormone therapy will be clarified by the analysis of patients’ quality of life questionnaires.

Additionally, evidence from our study showed that the site of metastases and inflammatory status appear to be associated, indeed, NLR and NLAR values were found to increase in patients with bone localizations, and, although not significant, an increasing trend in PLR was observed as well. As bone localizations are linked to a lower response rate than lymph node-only sites, this outcome is consistent with the well-known fact that a higher inflammatory status results in a worse prognosis. These results are in line with the study from Zhang et al. found a significant increase in NLR values and PLR in PCa patients with bone metastases [47]. Several studies and a meta-analysis showed that inflammatory biomarkers such as NLR were associated with poor OS, PFS, and recurrence-free survival in patients with PCa [48] and for patients with advanced PCa [49] elevated pretreatment NLR is significantly related to a worse prognosis. Correlation with oncological outcomes in future publications on our cohort will shed light on the prognostic power of the inflammatory biomarkers in oligometastatic patients harbouring a lower burden of disease.

When patients were divided into quartiles according to the time from surgery to oligorecurrence, 38% of the patients in the fourth quartile, with a median time to oligorecurrence > of 6.7 years, experienced a higher increase in the CONUT score. The CONUT score is used to assess the patients’ preoperative nutritional status and in a recent publication by Zhang et al. [40] CONUT score resulted as an independent prognostic factor significantly associated with PSA progression-free survival in oligometastatic PCa patients. Since the patients with a longer time from surgery to enrollment had a major increase in CONUT value this may be linked to a higher median age for these patients so they encountered a worsening in their nutritional condition. In addition, given the literature evidence, these patients may be closely monitored as patients with a higher risk of recurrence. As stated above, potential correlations with oncological outcomes are being investigated and will be the subject of future publications related to the RADIOSA trial.

The present study is not exempt from limitations, first and foremost of which is the limited number of patients and the paucity of patients (*n* = 47) with a minimum follow-up of 1 year. Nevertheless, this cohort represents one of the largest and most homogeneous populations in this setting.

To conclude, a reliable biological characterization of the oligometastatic state in prostate cancer still seems far from being achieved. The nutritional and inflammatory parameters examined in the current study do not seem to represent valuable candidates for possible use in clinical decision making in our cohort of patients. The presented data are going to be implemented with mutational landscape data from ctDNA of the enrolled patients for which next-generation sequencing (NGS) analyses are currently ongoing. These analyses will hopefully offer a valuable piece of evidence to stratify oligometastatic patients according to whether they benefit from more or less aggressive therapies.

## Figures and Tables

**Table 1:** Summary of patient cohort characteristics. Abbreviations: BMI (Body mass index); LDH (Lactate dehydrogenase); (NLR) Neutrophil-to-lymphocyte ratio; NLRAR (NLR–albumin ratio); HALP (Hemoglobin-albumin-lymphocyte-platelet index); PLR (Platelet-to-lymphocyte ratio); PNI (Prognostic Nutritional Index); (NRI) Nutrition Risk Index.

	Whole Cohort (*n* = 88)	ARM A (*n* = 45)	ARM B (*n* = 43)
**Variable**	**Median (IQR)**		
Age (years)	69 (64–75)	68 (63–74)	70 (66–76)
Height (cm)	172 (170–178)	173 (169–178)	172 (170–178)
Weight (kg)	81 (72–91)	82 (70–91)	81 (75–92)
BMI (kg/m²)	26.9 (24.69–29.24)	26.8 (24.2–29.5)	27.4 (25.1–29.2)
Time from surgery to oligorecurrence (months)	42 (28–81)	40 (27–79)	42 (32–88)
Time from last active treatment to oligorecurrence (months)	36 (17–64)	33 (17–58)	38 (19–69)
Neutrophils (10^3^/µlitro)	3.9 (3.4–5.1)	4.0 (3.4–5.0)	3.9 (3.5–5.5)
Lymphocites (10^3^/µlitro)	1.5 (1.3–1.9)	1.4 (1.3–1.8)	1.5 (1.2–2.0)
Platelets (10^3^/µlitro)	214 (184–246)	218 (189–244)	214 (177–246)
Hemoglobin (g/dL)	14.9 (14.3–15.7)	14.9 (14.5–15.5)	14.9 (14.2–15.8)
Testosterone (ng/mL)	3.8 (3.2–4.9)	3.6 (3.0–4.7)	4.0 (3.4–5.6)
Cholesterol (ml/dL)	193 (180–218)	194 (186–219)	193 (173–215)
Albumin (g/dL)	4.3 (4.2–4.5)	4.3 (4.2–4.5)	4.3 (4.2–4.4)
Glicemia (mg/dL)	96 (89–106)	98 (89–106)	95 (89–106)
LDH (mU/mL)	177 (157–201)	177 (159–208)	177 (156–194)
NLR	2.6 (2.1–3.4)	2.6 (2.2–3.5)	2.7 (2.0–3.2)
NLRAR	0.06 (0.05–0.08)	0.06 (0.05–0.08)	0.06 (0.05–0.07)
HALP	46 (34–67)	44 (33–63)	50 (36–70)
PLR	144 (101–186)	145 (106–194)	134 (95–180)
PNI	51 (49–53)	51 (49–53)	51 (49–54)
NRI	115 (111–120)	114 (110–121)	117 (111–120)
	**Counts (%)**		
**Lesion site**			
Lymphnode(s)	56	27	29
Bone	32	18	14
**CONUT SCORE**			
0	27	14	13
1	33	18	15
2	13	6	7
3	10	4	6
4	2	1	1
Missing	3	2	1

**Table 2:** Summary of biomarker levels at baseline, three months after SBRT, and overall change when patients were stratified by treatment arm. *p* values refer to comparison of change (3 months after SBRT—baseline, in bold) between arms.

	ARM A Median (IQR)			ARM B Median (IQR)			
	Baseline	3 Months FU	Change	Baseline	3 Months FU	Change	*p* Value ARM A vs. ARM B
Cholesterol (mL/dL)	194 (186–219)	208 (176–229)	**−1 (−22–18)**	193 (173–215)	207 (191–235)	**15 (1–29)**	**0.005**
Albumin (g/dL)	4.3 (4.2–4.5)	4.4 (4.2–4.5)	**0.0 (−0.1–0.1)**	4.3 (4.2–4.4)	4.2 (4.1–4.4)	**−0.1 (−0.2–0)**	**0.024**
Glicemia (mg/dL)	98 (88–106)	99 (86–107)	**0 (−4–5)**	95 (89–105)	99 (89–106)	**1 (−5–9)**	0.542
LDH (mU/mL)	177 (159–208)	181 (166–201)	**4 (−9–25)**	177 (156–193)	188 (168–204)	**16 (4–27)**	0.174
NLR	2.6 (2.2–3.5)	2.7 (2.06–3.7)	**0.1 (−0.5–0.4)**	2.7 (2.0–3.2)	2.4 (1.9–3.1)	**−0.2 (−0.7–0.1)**	0.141
NLRAR	0.06 (0.05–0.08)	0.06 (0.05–0.08)	**0.00 (−0.01–0.01)**	0.06 (0.05–0.07)	0.06 (0.05–0.07)	**0.00 (−0.02–0.01)**	0.369
HALP	43.9 (33.2–63.1)	44.0 (30.3–60.1)	**0.2 (−7.9–7.4)**	49.6 (35.7–70.1)	43.3 (29.3–57.5)	**−4.1 (−11.2–−0.5)**	0.067
PLR	145.3 (106.4–194.9)	145.4 (113.8–197.1)	**−0.1 (−19.7–16.2)**	133.7 (94.8–179.8)	140.4 (100.7–184.1)	**2.5 (−10.0–11.1)**	0.829
PNI	50.9 (48.7–53.3)	51.0 (48.4–52.4)	**0.0 (−2.0–1.7)**	51.3 (49.4–53.7)	49.9 (47.6–53.1)	**−1.1 (−3.4–0.8)**	0.132

Abbreviations: LDH (Lactate dehydrogenase); (NLR) Neutrophil-to-lymphocyte ratio; NLRAR (NLR–albumin ratio); HALP (Hemoglobin-albumin-lymphocyte-platelet index); PLR (Platelet-to-lymphocyte ratio); PNI (Prognostic Nutritional Index).

**Table 3:** Summary of biomarkers level at baseline, 1 year after SBRT and overall change when patients were stratified by treatment arm. *p* values refer to comparison of change (1 year after SBRT—baseline, in bold) between arms.

	ARM A Median (IQR)			ARM B Median (IQR)			
	Baseline	1 Year FU	Change	Baseline	1 Year FU	Change	*p* Value ARM A vs. ARM B
Testosterone (ng/mL)	3.6 (3.0–4.7)	4.2 (3–4.9)	**0.4 (−0.3–0.9)**	4.0 (3.4–5.6)	3.8 (2.1–5.5)	**0.0 (−2.3–0.8)**	0.204
Cholesterol (mL/dL)	194 (186–219)	192 (158–222)	**−1 (−15–14)**	193 (173–215)	194 (170–216)	**−2 (−20–5)**	0.945
Glicemia (mg/dL)	98 (88–106)	95 (86–109)	**−1 (−5–6)**	95 (89–105)	96 (89–107)	**−2 (−6–14)**	0.936
LDH (mU/mL)	177 (159–208)	179 (168–200)	**1 (−13–13)**	177 (156–193)	172 (149–193)	**−3 (−11–8)**	0.683

Abbreviations: LDH (Lactate dehydrogenase).

**Table 4:** Summary of biomarkers level at baseline, three months and overall change when patients were stratified by site of metastasis. *p* values refer to comparison of change (3 months after SBRT—baseline, in bold) between arms.

	Site of Metastases = Lymphnodal			Site of Metastases = Bone			
	Baseline	3 Months FU	Change	Baseline	3 Months FU	Change	*p* Value
Cholesterol (mL/dL)	191 (178–214)	210 (179–230)	**13 (−7–26)**	203 (186–227)	205 (190–233)	**1 (−14–18)**	0.174
Albumin (g/dL)	4.3 (4.2–4.5)	4.3 (4.1–4.5)	**−0.1 (−0.2–0.1)**	4.3 (4.2–4.5)	4.3 (4.2–4.5)	**0.0 (−0.1–0.1)**	0.889
Glicemia (mg/dL)	95 (88–106)	97 (86–106)	**1 (−5–5)**	100 (91–105)	100 (91–108)	**0 (−5–7)**	0.865
LDH (mU/mL)	178 (156–203)	181 (168–201)	**12 (−7–26)**	169 (159–193)	190 (168–206)	**8 (−6–27)**	0.936
NLR	2.7 (2.1–3.4)	2.4 (1.9–3.4)	**−0.2 (−0.7–0.2)**	2.6 (2.1–3.2)	2.7 (2.1–3.8)	**0.1 (−0.3–0.6)**	**0.020**
NLRAR	0.06 (0.05–0.08)	0.05 (0.05–0.08)	**−0.01 (−0.02–0.01)**	0.06 (0.05–0.07)	0.06 (0.05–0.09)	**0.00 (−0.01–0.01)**	**0.049**
HALP	47.5 (33.8–64.3)	46.4 (31.0–63.0)	**−2.1 (−8.4–4.4)**	44.3 (34.9–67.5)	39.7 (28.9–52.9)	**−4.5 (−10.7–1.1)**	0.226
PLR	137.6 (106.5–190.8)	138.2 (101.0–184.1)	**−1.6 (−14.7–9.1)**	148.0 (100.0–180.5)	159.7 (128.9–192.9)	**5.9 (−2.8–27.7)**	0.061
PNI	51.7 (49.3–53.4)	51.3 (48.4–52.6)	**−0.8 (−2.8–1.3)**	50.6 (49.1–53.0)	49.5 (47.6–51.9)	**−0.7 (−2.2–0.6)**	0.912

Abbreviations: LDH (Lactate dehydrogenase); (NLR) Neutrophil-to-lymphocyte ratio; NLRAR (NLR–albumin ratio); HALP (Hemoglobin-albumin-lymphocyte-platelet index); PLR (Platelet-to-lymphocyte ratio); PNI (Prognostic Nutritional Index).

## Data Availability

Data are available data is unavailable due to privacy or ethical restrictions.

## Abbreviations

ADT	Androgen deprivation therapy
BCR	Biochemical recurrence
BED	Biologically Effective Dose
BMI	Body mass index
CONUT	Controlling nutritional status
HALP	Hemoglobin-albumin-lymphocyte-platelet
IQR	Inter quartile range
LDH	Lactate dehydrogenase
LHRH	Luteinizing hormone-releasing hormone
MDT	Metastasis-directed therapy
NGS	Next Generation Sequencing
NLR	Neutrophil-to-lymphocyte ratio
NLRAR	NLR–albumin ratio
NRI	Nutrition Risk Index
PCa	Prostate cancer
PLR	Platelet-to-lymphocyte ratio
PSMA-PET	Prostate-specific membrane antigen—positron emission tomography
PNI	Prognostic Nutritional Index
SBRT	Stereotactic body radiation therapy
